# Drug therapy problems identified among patients receiving antiretroviral treatment in a HIV clinic: a prospective study in North Central, Nigeria

**DOI:** 10.11604/pamj.2021.40.233.28160

**Published:** 2021-12-16

**Authors:** Roseline Iberi Aderemi-Williams, Sabina Onyinye Nduaguba, Eric Monday Akoji, Patricia Uche Ogbo, Isaac Okoh Abah

**Affiliations:** 1 Department of Clinical Pharmacy and Biopharmacy, Faculty of Pharmacy, University of Lagos, Idiaraba, Lagos State, Nigeria,; 2 Pharmaceutical Systems and Policy Department, West Virginia University School of Pharmacy, Morgantown, West Virginia, USA,; 3 West Virginia University Cancer Institute, Morgantown, West Virginia, USA,; 4 Pharmacy Department, General Hospital Ankpa, Kogi State, Nigeria,; 5 Pharmacy Department, Jos University Teaching Hospital (JUTH), Plateau State, Nigeria

**Keywords:** Medication therapy management, pharmaceutical services, drug interactions, patient care management, antiretroviral therapy, HIV

## Abstract

**Introduction:**

despite improved life expectancy for people living with HIV (PLWH), aging, comorbidities, and associated drug treatment increase the risk for drug therapy problems (DTPs). We assessed pharmacists´ identification and resolution of DTPs among PLWH.

**Methods:**

a prospective study was conducted among PLWH aged ≥10 years (N=100) in a Nigerian HIV clinic. Trained pharmacists delivered a six-step intervention that included the establishment of patient-provider relationship, gathering and validation of patient´s data, identification of DTPs, intervention, outcome identification, and documentation. Descriptive statistics were used to examine data collected via a pharmaceutical care assessment tool.

**Results:**

in all, 215 DTPs were identified and classified as unnecessary drug therapy [27.4% (n=59)], non-adherence [21.9% (n=47)], needs additional drug therapy [16.7% (n=36)], adverse drug reaction [(14.0% (n=30)], wrong drug [(10.7% (n=23)], and dosage variation [n=20 (9.3%)]. Within each DTP class, the most common cause was addiction/recreational drug use [39.0% (n=23)], drug product not available [63.8% (n=30)], untreated condition(s) [61.1% (n=22)], undesirable effects [66.7% (n=20)], condition refractory to drug [34.8% (n=8)], and drug interaction [45.0% (n=9)], respectively. The most common interventions were medication information/recommendation to patients/prescribers (30.4%) and initiation of drug therapy (22.2%). Six-month resolution rate was 90% (n=194) with the most common outcomes being improvement in patient adherence [23.6% (n=50)], addition of a drug [18.9% (n=40)], and reduction in drug overuse [15.6% (n=33)].

**Conclusion:**

pharmacists´ intervention resulted in 90% resolution of detected DTPs, implying that pharmacists are crucial in improving antiretroviral treatment outcomes.

## Introduction

Nigeria, like many other countries around the world has been affected by the HIV epidemic. The first case was diagnosed in 1986 [1]. By 2003, HIV prevalence in Nigeria was at 5.4% with the number of people living with HIV estimated to be 3.6 million [2]. This placed Nigeria third among countries with the highest burden attributable to HIV after South Africa and India [3]. Since then, HIV prevalence rate in Nigeria has declined. Most recent data showed that the prevalence of HIV in Nigeria was 1.5% in 2018 [4]. Currently, about 2 million people are living with HIV in Nigeria [5]. Kogi state located in middle-belt (North Central) Nigeria is reported to be one of the states currently with a prevalence rate between 0.7 - 1.0% [4]. People living with HIV (PLWH) are living longer with life expectancy close to the general population in many countries, thanks to improved access to effective antiretroviral treatment [6-9]. Aging, coupled with higher prevalence of HIV and non-HIV comorbidities, imply that many PLWH may require multiple medications, not only for managing HIV infection but for other chronic conditions [10-14]. These increase the risk for drug-related problems which could range from untreated indication to improper drug selection, too many or too few drugs, drug interactions, nonadherence, and adverse drug reaction [15]. Such problems are associated with significant morbidity and mortality and increased healthcare resource utilization and costs [16-18]. Drug-related problems and their associated costs have, however, been shown to be preventable through the provision of pharmaceutical services [16,17,19-21]. Pharmaceutical care has been defined as “the responsible provision of drug therapy to achieve definite outcomes that improve a patient´s quality of life” [22]. As practitioners of pharmaceutical care, pharmacists are responsible for meeting the drug therapy-related needs of their patients, including identifying and addressing actual and potential problems associated with medication use. With over 53% of PLWH in Nigeria on antiretroviral treatment and 42% virally suppressed [4,5], life expectancy and the prevalence of comorbid conditions are expected to be on the increase. The need for pharmacists in the management of patients with HIV in Nigeria is now greater than before. In this study, we aimed to assess the identification and resolution of drug-related problems and issues influencing their occurrence among patients receiving antiretroviral therapy in an antiretroviral treatment clinic located in the North Central region of Nigeria.

## Methods

**Study design and setting:** a prospective study was conducted at a secondary health facility, General Hospital located in Kogi state, Nigeria. The HIV clinic within the general hospital is the second treatment center in the state and was established in 2007 as part of Nigeria´s national response to the HIV/AIDS pandemic. Patients with HIV can receive comprehensive care at the HIV clinic, eliminating the need for them to attend the hospital's outpatient department. At the time services commenced in 2007, 80-85% of prescription needs were covered for free. The study was carried out between April and October 2013.

**Study population:** patients eligible for the study were persons living with HIV who received care at the HIV clinic. To be included in the study, patients had to have a confirmed diagnosis of HIV, be receiving antiretroviral therapy, be at least 10 years old, and have a history of a drug therapy problem. Patients with mental health disorders were excluded. Based on the state HIV prevalence rate of 5.8% in 2011 [23], a degree of accuracy set at 95%, and expected attrition of 20%, the required sample size for the study was determined to be 100. Recruitment was done using convenience sampling.

**Intervention:** an in-person pharmaceutical care intervention was provided individually to participating patients by pharmacy staff in a space dedicated to counseling within the pharmacy. To ensure consistency in the administration of the intervention, participating pharmacy staff underwent a two-day training on the pharmaceutical care plan and the use of a pharmaceutical care assessment tool. The framework for the intervention was based on the nine-step process by Cipolle *et al*. (1998) and delineated into six steps [24]. First, the pharmacist establishes a therapeutic relationship with the patient through a mutual introduction and asking about the patient´s health condition. Second, the pharmacist collects data from the patient and validates the obtained information against medical records and extracts additional information from the records. The information includes patient name and address, weight, age, sex, history of allergies, laboratory data on CD4 count, packed cell volume (PCV), and liver function test, prescribed medications, previous medications, side effects, adherence to medical appointments, and medication adherence including taking medication with food, drink, or beverage and taking a higher or lower dose than what was prescribed. In the third step, the pharmacist evaluates the data collected and identifies drug therapy problems by verifying the patient´s prescription and checking for prescription anomalies such as prescription errors, food-drug interactions, drug-drug interactions, and drug-disease interactions. The pharmacist also asks about any other health complaints not disclosed to the prescriber, use of other medications, need for other prescribed medication that has not been provided, financial challenges with purchasing medication, the experience of any abnormal or unusual effects as a result of taking their medication, and whether they keep their appointment dates.

Following the evaluation, the fourth step was an intervention. The pharmacist intervention involved taking one or more of the following actions: contact prescriber or other health personnel for clarification on a prescription error or drug change, suggest to prescriber appropriate measures that could resolve an identified drug therapy problem, provide recommended medication information to the patient, discuss event with the patient or caregivers, counsel patient to return to the facility in the event of side effects/adverse drug reactions, initiate drug therapy as prescribed, implement change in drug therapy based on interaction with the prescriber, provide adherence counseling, and/or counsel patient against overuse of drug therapy or on non-pharmacological measures to minimize avoidable side effects. The fifth step was to identify the proposed outcome from the intervention which could be one of the following: pharmacist changes the medication following consultation with prescriber; pharmacist asks the patient to stop a medication; addition of a medication; patient´s adherence to drug therapy improves; patient´s adherence to non-drug therapy improves; or patient reduces overuse of drug therapy. Finally, the pharmacist documents the intervention, the medication dispensed, any side effect/adverse drug reaction being experienced by the patient, the patient´s next appointment, patient´s prescription order form, and all laboratory data relevant in assessing outcomes of therapy. Participating patients were seen monthly for a total of six sessions. Each session lasted about 30 minutes. The pharmaceutical care assessment tool was used at each visit and intervention provided when any drug-related problem was identified. To minimize loss to follow-up, participants who missed their appointments were contacted by phone or home visitation by field trackers.

**Data collection:** a pharmaceutical care assessment tool designed for the documentation of pharmaceutical care services in HIV/AIDS was used by the participating pharmacists to document the encounter with the patient. Using information from documentation of the pharmaceutical care assessments provided over the course of the study, an analytical dataset was created with the unit of observation being the identified drug-related problems.

**Definitions:** identified drug therapy problems and their causes were coded and classified using the cipolle method [24]. The cipolle method classifies drug therapy problems into: unnecessary drug therapy, need for additional drug therapy, ineffective drug, dosage too low, adverse drug reaction, dosage too high, and nonadherence. We merged problems related to dosage too low and dosage too high into dosage variation. Reasons for each diphtheria-pertussis-tetanus vaccine (DTP) was abstracted from the pharmaceutical care assessment tool and categorized based on identified themes. The actions taken to address each drug-related problem were categorized into seven intervention strategies: 1) no intervention taken; 2) contacted the prescriber or other health personnel to clarify error; 3) medication not dispensed after clarification and recommendation rejected; 4) medication information/recommendation provided to patients and prescribers; 5) counseled patients or caregiver about event; 6) drug therapy initiated after consultation with prescriber; and 7) drug therapy changed after clarification and recommendation accepted. The outcome of intervention was also categorized into seven themes: 1) medication stopped; 2) medication changed; 3) dose changed; 4) medication added; 5) patient adherence to medication therapy increased; 6) patient overuse of drug therapy decreased; 7) patient adherence to non-drug therapy increased.

**Statistical analysis:** the research data was analyzed descriptively using frequency and percentages with pairwise deletion of missing data. Identified Analysis was conducted using IBM SPSS statistics for Window version 20.0 (IBM Corp, Armonk, New York, USA).

**Ethical considerations:** for participants aged below 18 years, assent and parental consent was obtained while signed informed consent was obtained from patients 18 years and above who agreed to participate in the study. The study was approved by the Kogi State Hospital Management Institutional Review Board.

## Results

**Patient characteristics:** of the 970 patients that attended the clinic within the six months of the study, 10% of those (n=100) patients who experienced drug therapy problem(s) were invited to participate in the study. Seventy-six percent (76%)(n=76) of the 100 study participants were female, and 93% (n=93) were 15 years or older.

**Identified drug therapy problems and their classification:** a total of 215 drug-related problems were identified (mean=2.2). Table 1 shows the classification of the identified drug therapy problems based on the Cipolle framework. In order of decreasing frequency, the identified DTPs included 59 (27.4%) unnecessary drug therapy, 47 (21.9%) non-adherence, 36 (16.7%) needs additional drug therapy, 30 (14.0%) adverse drug reaction, 23 (10.7%) wrong drug, and 20 (9.3%) dosage variation.

**Table 1:** classes of drug therapy problems and resolution status at 6 months (N=100)

Class of drug therapy problema	N (%)	Resolution status n (row percent)	
		Resolved	Unresolved
Unnecessary drug therapy	59 (27.4)	52 (88.1)	7(11.9)
Non-adherence	47 (21.9)	41(87.2)	6 (12.8)
Need additional drug therapy	36 (16.7)	32 (88.9)	4 (11.1)
Adverse drug reaction	30 (14.0)	28 (93.3)	2 (6.7)
Wrong drug	23 (10.7)	22 (95.6)	1(4.4)
Dosage variation	20 (9.3)	19 (95.0)	1 (5.0)
TOTAL	215 (100.0)	194 (90.2)	21 (9.8)

a The drug therapy problems are classified using the Cipolle framework

**Causes of identified drug therapy problems according to the Cipolle framework:** the causes of each class of drug therapy problem are presented in Table 2. The most common causes of unnecessary drug therapy, non-adherence, need for additional drug therapy, adverse drug reaction, wrong drug, and dosage variation were addiction/recreational drug use [39.0% (n=23)], drug product not available [63.8% (n=30)], untreated conditions [61.1% (n=22)], undesirable effects [66.7% (n=20)], condition refractory to a drug [34.8% (n=8)], and drug interaction [45.0% (n=9)] respectively.

**Table 2:** specific causes of drug therapy problems by problem class (N=100)

Causes^a^	Frequency	Percentage (%)
Unnecessary drug therapy (N=59)		
Addiction/recreational drug use	23	39.0
Duplicate therapy	12	20.3
No medical indication	10	16.9
Treating avoidable adverse drug reaction	9	15.3
Non-drug therapy more appropriate	5	8.5
Non-Adherence (N=47)		
Drug product not available	30	63.8
Cannot afford drug product	11	23.4
Cannot swallow/administer drug	4	8.5
Does not understand instruction	1	2.1
Patient prefers not take drug	1	2.1
Needs additional drug therapy (N=36)		
Untreated conditions	22	61.1
Prophylactic therapy	10	27.8
Synergistic therapy	4	11.1
Adverse drug reaction (N=30)		
Undesirable effects	20	66.7
Allergic reaction	4	13.3
Unsafe drug for patient	4	13.3
Incorrect administration	2	6.7
Wrong drug (N=23)		
Condition refractory to drug	8	34.8
More effective drug available	7	30.4
Contra-indication present	3	13.0
Drug not indicated for condition	3	13.0
Dosage form inappropriate	2	8.7
Dosage variation (N=20)		
Drug interaction	9	45.0
Dosage too low	4	20.0
Frequency inappropriate	3	15.0
Dosage too high	2	10.0
Duration inappropriate	2	10.0

a causes of the drug threrapy problems are categorized using the Cipolle framework

**Figure 1:**
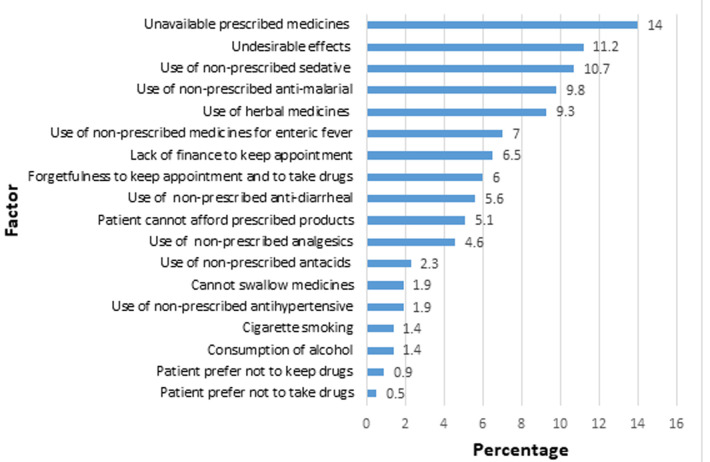
factors influencing the occurrence of a drug-related problem

**Reasons Identified by the pharmacist for the occurrence of drug therapy problems:** eighteen reasons were identified by the participating pharmacists as influencing the occurrence of the drug therapy problems (Figure 1). The most common reasons were: unavailable prescribed medicines [14.0% (n=30)], undesirable effects [11.2% (n=24)], use of unprescribed sedative [10.7% (n=23], use of unprescribed antimalarial [9.8% (n=21)], and use of herbal remedies [9.3% (n=20)].

**Interventions provided by pharmacist:** the most common interventions provided by the pharmacists were the provision of medication information or recommendation to patients and prescribers [30.4% (n=60)] and initiation of drug therapy after consultation with the prescriber [22.2% (n=41)] (Table 3).

**Table 3:** pharmacist’s intervention strategies and outcomes of interventions (N=100)

Intervention strategies	Frequency	Percentage (%)
Medication information/recommendation provided to patients and prescribers	60	28.6
Drug therapy initiated after consultation with prescriber	41	19.5
Counseled patients or caregiver about event	40	19.0
Contacted the prescriber or other health personnel to clarify error	30	14.3
Drug therapy changed after clarification and recommendation accepted	30	14.3
Medication not dispensed after clarification and recommendation rejected	9	4.3
No intervention taken	0	0.0
TOTAL	210	100.0
Outcomes of Interventions	Frequency (n)	Percentage (%)
Patient adherence to medication therapy increased	50	23.6
Medication added	40	18.9
Patient overuse of medication therapy decreased	33	15.6
Medication changed	30	14.2
Medication stopped	27	12.7
Patient adherence to non-drug therapy increased	19	9.0
Dose changed	13	6.1
Total	212	100.1^a^

a Does not add up to 100.0% due to rounding

**Resolution and outcome of intervention:** ninety percent (n=194) of the drug therapy problems were resolved by six months with the most common outcomes being an improvement in patient adherence [23.6% (n=50)], the addition of a drug [18.9% (n=40)], and reduction in patient overuse of drug therapy [15.6% (n=33)] (Table 3).

## Discussion

In this study, we identified and assessed causes of drug therapy problems through the provision of pharmaceutical care to PLWH on ART. We found an average of two drug therapy problems per patient with unnecessary drug therapy being the most frequent problem class. Unavailable prescribed drugs, unwanted side effects, and the use of non-prescribed medicines were the three most common causes of drug therapy issues. Over 90% of drug therapy problems were resolved with the most frequent outcome being improved adherence. The identification of two drug therapy problems per patient is higher than other prospective studies which recorded less than one drug therapy problem per patient with HIV [25-27]. Our study may be different from these studies in that we recruited patients who already had a history of drug therapy problem making it more likely that we would find a drug therapy problem prospectively. This suggests that drug therapy problems may cluster among patients with high risk for drug therapy problems. For example, in a cross-sectional study, Kara *et al*. reported that the prevalence rate of polypharmacy was 30% among 181 outpatients in Turkey [28]. However, the 58 identified drug therapy problems were clustered among 45 patients. Similarly, Carcelero identified 60 drug therapy problems clustered among 41 patients in a study of 187 hospitalized patients [29]. Hence, identification of at-risk patients is an important first step to addressing drug therapy problems. Studies show that age, duration of antiretroviral therapy (ART), intensive ART, and polypharmacy are significant predictors of drug therapy problems among patients with HIV [28,30].

Unnecessary drug therapy, non-adherence, and need for additional drug therapy were the top three classes of drug therapy problems identified in our study, accounting for 66% of the identified problems. A similar study conducted in the North Central part of Nigeria recorded unnecessary drug therapy as the second most common problem after drug omission [31]. In a study conducted in Brazil, the most common intervention provided was to address or prevent non-adherence [32]. A study conducted in Turkey identified sub-optimal treatment and an untreated indication as the most common problems among outpatients with HIV [28]. Non-adherence is a drug therapy problem that requires the patient's cooperation to resolve, whereas the need for more therapy is a drug therapy problem that requires the prescriber's cooperation to resolve. Patients and their prescribers are therefore both important stakeholders for the successful provision of pharmaceutical care. Overall, we had a resolution rate of 90%, ranging from 88% to 96%, depending on the class of drug therapy problem. This agrees with several studies in the literature with a resolution rate ranging from 86% to 93% [28,29,31]. One exception was the study by Silveira *et al*. in which the resolution rate was 43% [25]. However, over 40% of the drug therapy problems they identified were related to ineffective response, which requires in-depth information of patient clinical history and knowledge of previous therapeutic failures. Again, this highlights the importance of the co-operation of providers caring for a patient in a multidisciplinary care team to coalesce information about a patient medical history to address complex health problems.

The use of unprescribed medicines accounted for over 50% of the reasons identified by the pharmacists for the occurrence of drug therapy problem. The medicines used without a prescription by participants in our study included sedatives, antimalarials, herbal medicines, medicines for enteric fever, antidiarrheals, analgesics, antacids, and antihypertensives. Self-medication is a common practice in Nigeria with the most commonly implicated medicines being analgesics, antimalarials, and antibiotics [33-35]. Although the World Health Organization encourages the reclassification of medicines from prescription-only status to over-the-counter status to enable self-medication [36], it should be noted that many prescription-only medicines, including antibiotics, are easily accessible without prescription in Nigeria, especially from patent medicine stores [33,37]. Further, patients being treated for chronic conditions like HIV may not be aware of potential hazards including drug-drug interactions associated with concomitant use of their prescribed medicines and medicines taken on their initiative [33,38,39]. Access to care and cost of care have been cited as major reasons to self-medicate [33-35,39]. As a factor influencing more than half of drug therapy problems identified in our study, the need to encourage patients with HIV to disclose to their providers at the HIV clinic when they self-medicate as their HIV providers are in a better position to advise on potential implications of concomitant use with ART, provide medical advice for their comorbidity, or assist with referrals. A limitation of our study was the absence of a control group to determine the effect of pharmaceutical care intervention. However, our goal of describing drug-related problems in PLWH receiving ART and resolving identified problems was achieved. Other studies have shown improvement in HIV-related clinical outcomes such as an increase in CD4 count with pharmacist intervention [32]. One strength of our study is that we identify the causes of drug therapy problem separately for each class which can help in the tailoring of interventions to address drug therapy problem based on predisposing factors that are unique to each class. We, however, recommend caution in generalizing our findings as participants already had a history of drug-related problems. Patients newly initiating therapy and those with less complex clinical profile may have fewer drug therapy problems than the average of two identified in our study.

## Conclusion

In the studied environment, drug therapy issues are common among PLWH taking ART. The intervention of pharmacists resulted in the resolution of 90% of the detected drug therapy problems, implying that pharmacists play a crucial role in improving ART treatment outcomes. The use of unprescribed medicines was the most prevalent reason influencing the occurrence of drug therapy problems among patients with HIV in our study. HIV care providers, particularly pharmacists, need to encourage disclosure of self-medication by their patients to allow for the identification and resolution of potential or actual drug therapy problems that may arise from contraindications, drug interactions, unnecessary treatment, duplicate therapy, or other inappropriate use.

### What is known about this topic

- *Due to improved access to treatment, people living with HIV (PLWH) are living longer;*
- Many PLWH may require multiple medications due to aging and comorbidities;
- *Through the provision of pharmaceutical care, pharmacists may identify potential or actual drug therapy problems among PLWH*.

### What this study adds

- *Pharmacists providing care to patients with HIV are able to identify and resolve most drug therapy problems;*
- *Use of unprescribed medicines is a prevalent reason influencing the occurrence of drug therapy problems among patients with HIV*.
